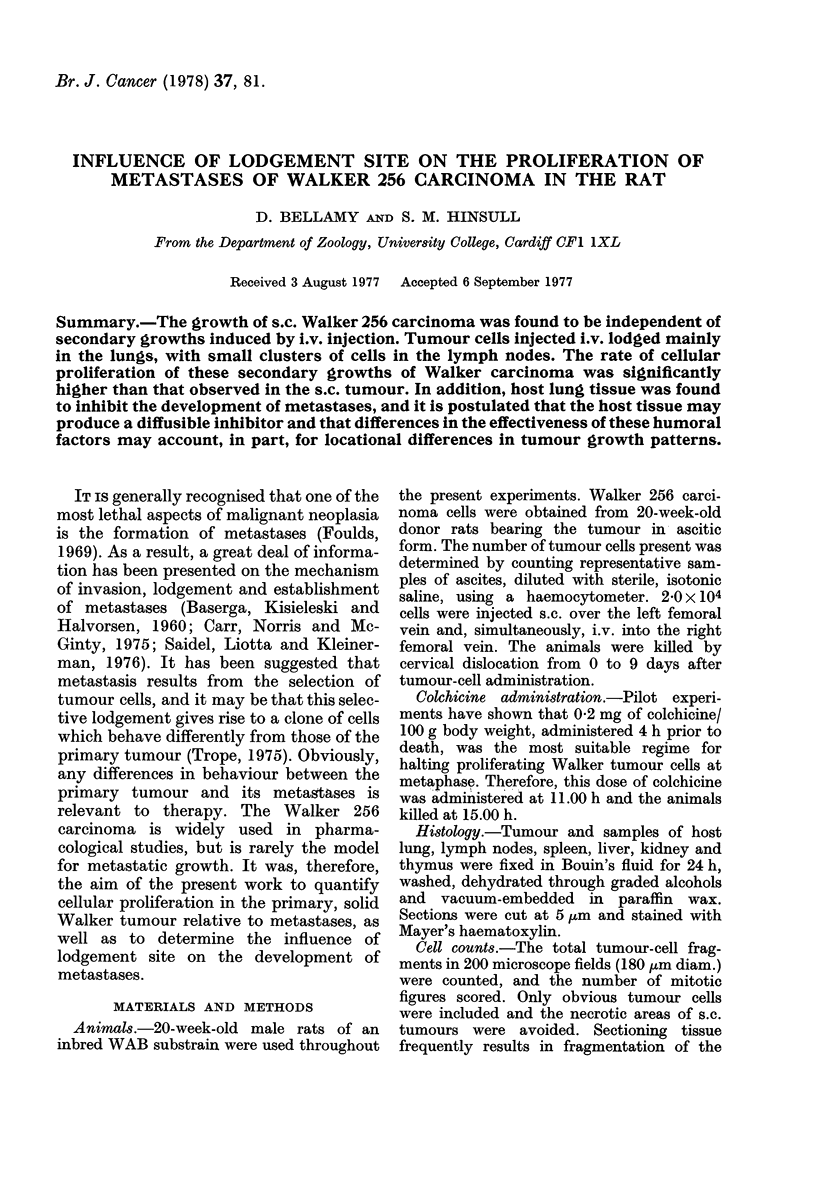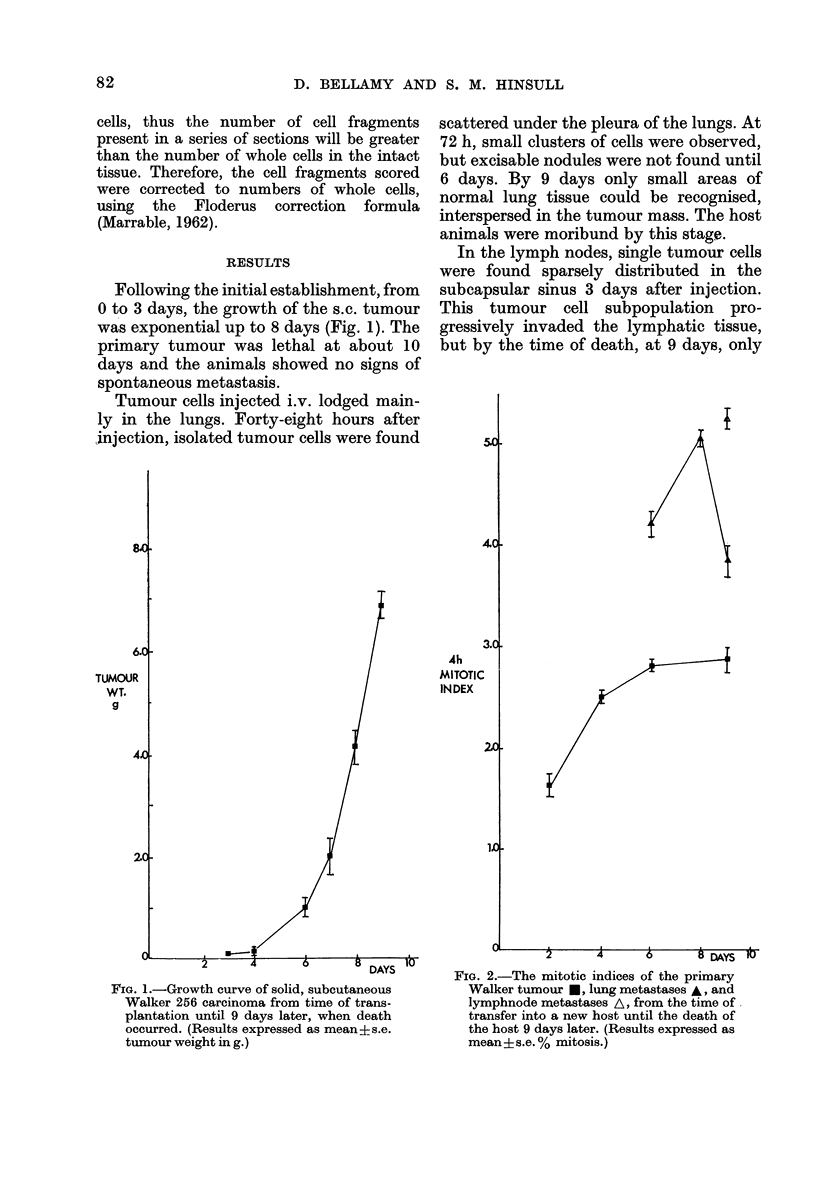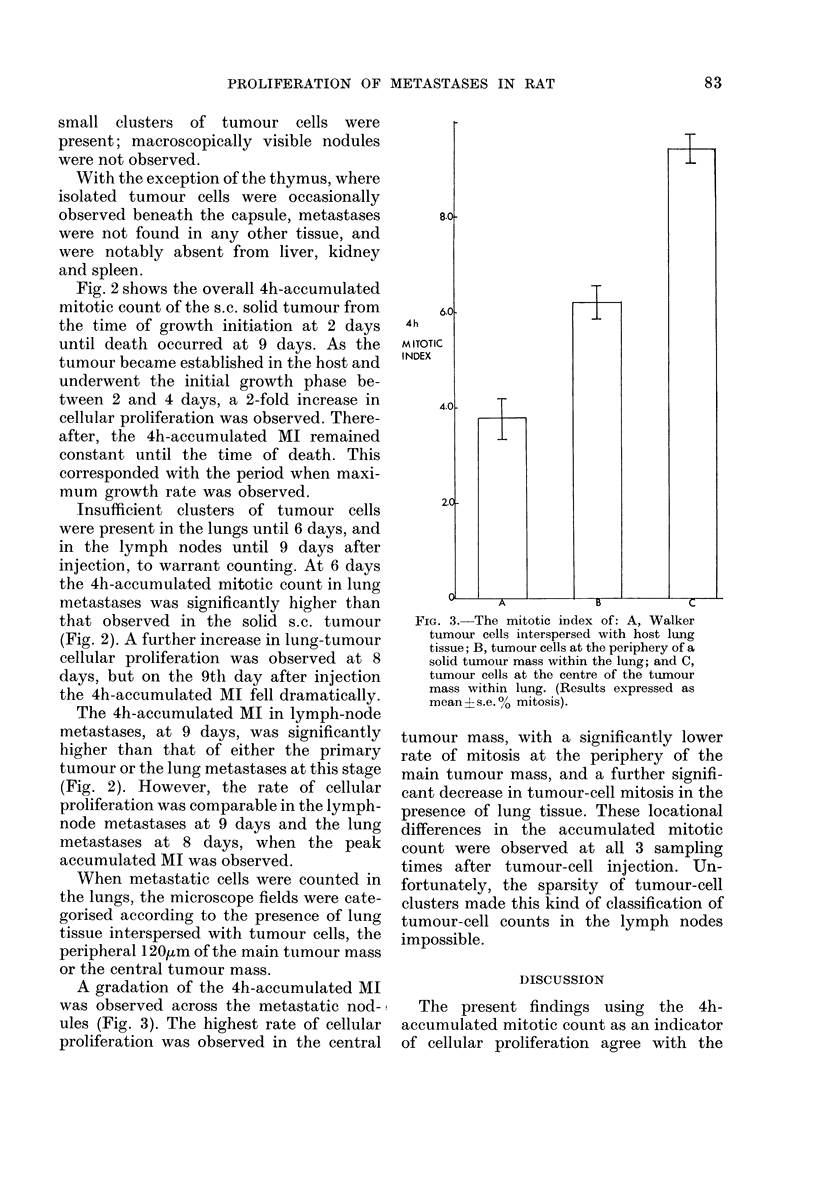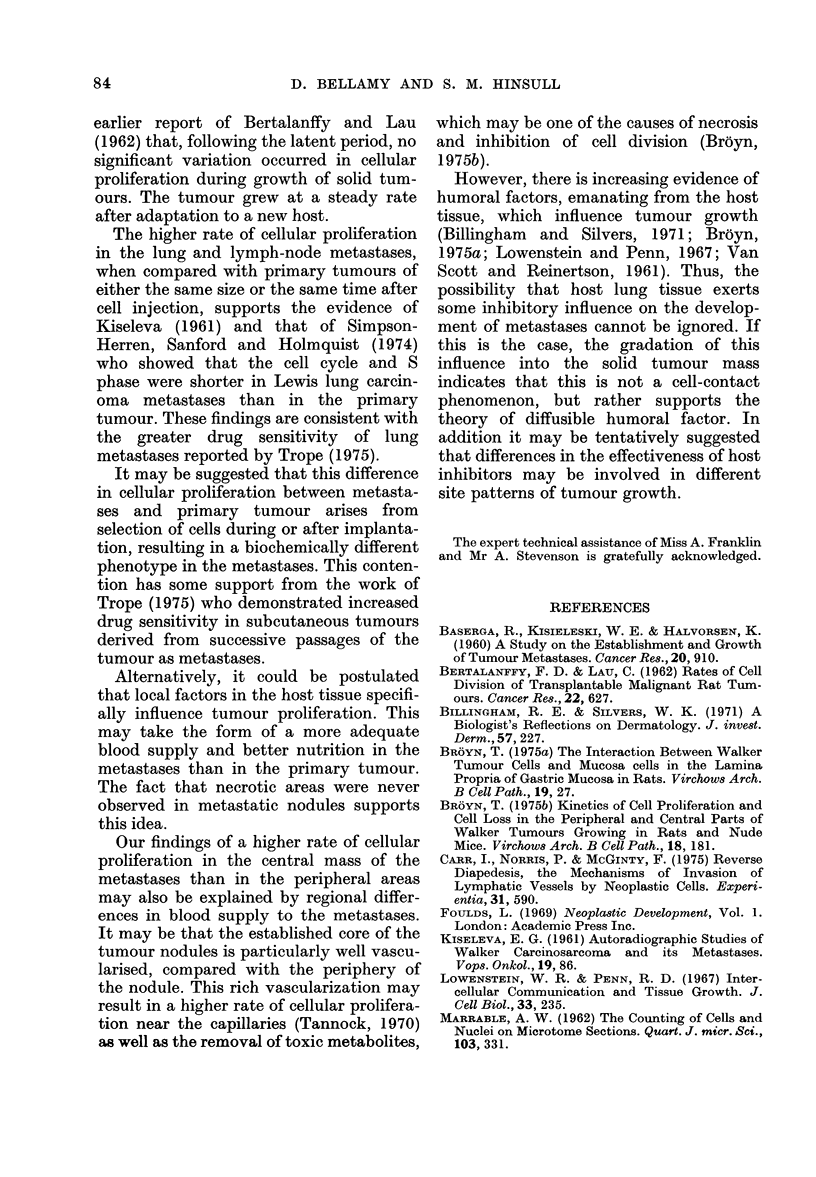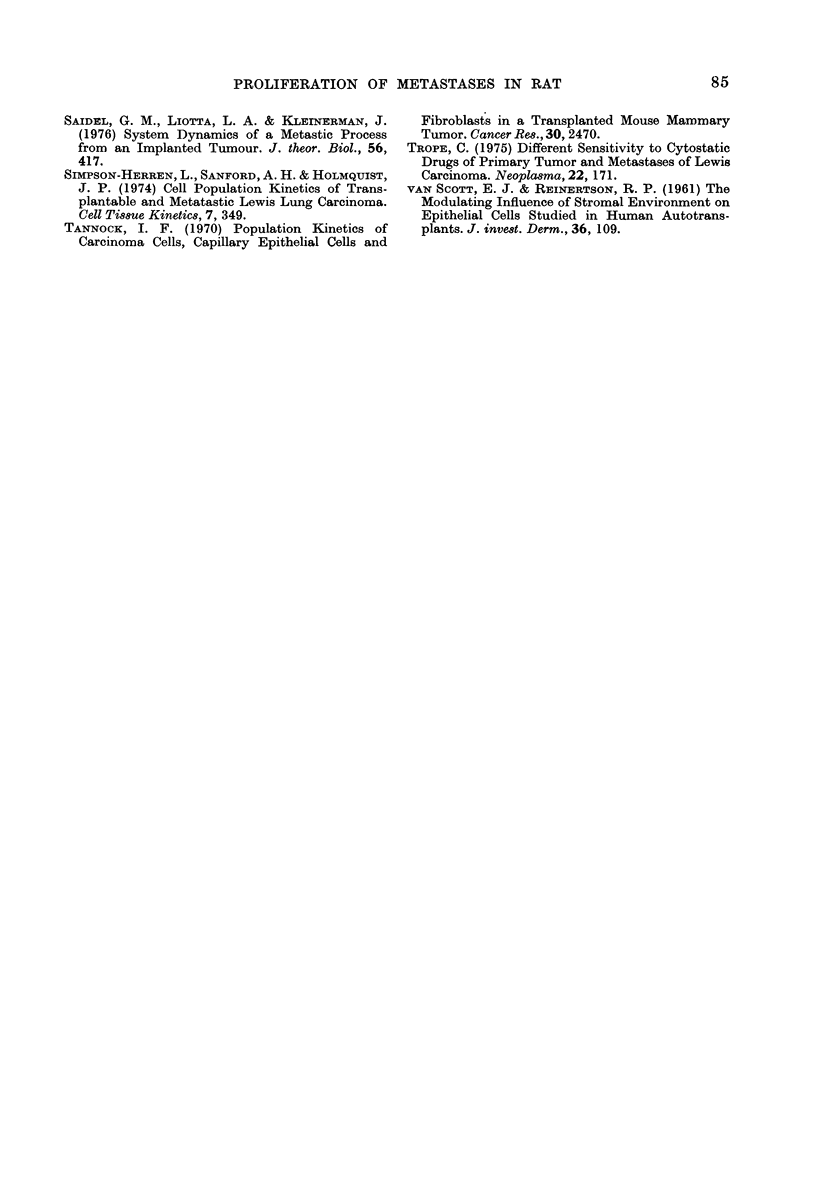# Influence of lodgement site on the proliferation of metastases of Walker 256 carcinoma in the rat.

**DOI:** 10.1038/bjc.1978.12

**Published:** 1978-01

**Authors:** D. Bellamy, S. M. Hinsull

## Abstract

The growth of s.c. Walker 256 carcinoma was found to be independent of secondary growths induced by i.v. injection. Tumour cells injected i.v. lodged mainly in the lungs, with small clusters of cells in the lymph nodes. The rate of cellular proliferation of these secondary growths of Walker carcinoma was significantly higher than that observed in the s.c. tumour. In addition, host lung tissue was found to inhibit the development of metastases, and it is postulated that the host tissue may produce a diffusible inhibitor and that differences in the effectiveness of these humoral factors may account, in part, for locational differences in tumour growth patterns.


*Br. J. Cancer* (1978) **37**, 81.

### INFLUENCE OF LODGEMENT SITE ON THE PROLIFERATION OF METASTASES OF WALKER 256 CARCINOMA IN THE RAT

D. BELLAMY AND S. M. HINSULL

*From the Department of Zoology, University College, Cardiff CF1 1XL*

Received 3 August 1977 Accepted 6 September 1977

**Summary.—The growth of s.c. Walker 256 carcinoma was found to be independent of secondary growths induced by i.v. injection. Tumour cells injected i.v. lodged mainly in the lungs, with small clusters of cells in the lymph nodes. The rate of cellular proliferation of these secondary growths of Walker carcinoma was significantly higher than that observed in the s.c. tumour. In addition, host lung tissue was found to inhibit the development of metastases, and it is postulated that the host tissue may produce a diffusible inhibitor and that differences in the effectiveness of these humoral factors may account, in part, for locational differences in tumour growth patterns.**

IT IS generally recognised that one of the most lethal aspects of malignant neoplasia is the formation of metastases (Foulds, 1969). As a result, a great deal of information has been presented on the mechanism of invasion, lodgement and establishment of metastases (Baserga, Kisieleski and Halvorsen, 1960; Carr, Norris and McGinty, 1975; Saidel, Liotta and Kleinerman, 1976). It has been suggested that metastasis results from the selection of tumour cells, and it may be that this selective lodgement gives rise to a clone of cells which behave differently from those of the primary tumour (Trope, 1975). Obviously, any differences in behaviour between the primary tumour and its metastases is relevant to therapy. The Walker 256 carcinoma is widely used in pharmacological studies, but is rarely the model for metastatic growth. It was, therefore, the aim of the present work to quantify cellular proliferation in the primary, solid Walker tumour relative to metastases, as well as to determine the influence of lodgement site on the development of metastases.

### MATERIALS AND METHODS

*Animals.*—20-week-old male rats of an inbred WAB substrain were used throughout the present experiments. Walker 256 carcinoma cells were obtained from 20-week-old donor rats bearing the tumour in ascitic form. The number of tumour cells present was determined by counting representative samples of ascites, diluted with sterile, isotonic saline, using a haemocytometer. $2{\cdot}0 \times 10^4$ cells were injected s.c. over the left femoral vein and, simultaneously, i.v. into the right femoral vein. The animals were killed by cervical dislocation from 0 to 9 days after tumour-cell administration.

*Colchicine administration.*—Pilot experiments have shown that 0·2 mg of colchicine/100 g body weight, administered 4 h prior to death, was the most suitable regime for halting proliferating Walker tumour cells at metaphase. Therefore, this dose of colchicine was administered at 11.00 h and the animals killed at 15.00 h.

*Histology.*—Tumour and samples of host lung, lymph nodes, spleen, liver, kidney and thymus were fixed in Bouin's fluid for 24 h, washed, dehydrated through graded alcohols and vacuum-embedded in paraffin wax. Sections were cut at 5 $\mu$m and stained with Mayer's haematoxylin.

*Cell counts.*—The total tumour-cell fragments in 200 microscope fields (180 $\mu$m diam.) were counted, and the number of mitotic figures scored. Only obvious tumour cells were included and the necrotic areas of s.c. tumours were avoided. Sectioning tissue frequently results in fragmentation of the cells, thus the number of cell fragments present in a series of sections will be greater than the number of whole cells in the intact tissue. Therefore, the cell fragments scored were corrected to numbers of whole cells, using the Floderus correction formula (Marrable, 1962).

### RESULTS

Following the initial establishment, from 0 to 3 days, the growth of the s.c. tumour was exponential up to 8 days (Fig. 1). The primary tumour was lethal at about 10 days and the animals showed no signs of spontaneous metastasis.

Tumour cells injected i.v. lodged mainly in the lungs. Forty-eight hours after injection, isolated tumour cells were found scattered under the pleura of the lungs. At 72 h, small clusters of cells were observed, but excisable nodules were not found until 6 days. By 9 days only small areas of normal lung tissue could be recognised, interspersed in the tumour mass. The host animals were moribund by this stage.

In the lymph nodes, single tumour cells were found sparsely distributed in the subcapsular sinus 3 days after injection. This tumour cell subpopulation progressively invaded the lymphatic tissue, but by the time of death, at 9 days, only

FIG. 1.—Growth curve of solid, subcutaneous Walker 256 carcinoma from time of transplantation until 9 days later, when death occurred. (Results expressed as mean $\pm$ s.e. tumour weight in g.)

FIG. 2.—The mitotic indices of the primary Walker tumour ■, lung metastases ▲, and lymphnode metastases △, from the time of transfer into a new host until the death of the host 9 days later. (Results expressed as mean $\pm$ s.e. % mitosis.)

small clusters of tumour cells were present; macroscopically visible nodules were not observed.

With the exception of the thymus, where isolated tumour cells were occasionally observed beneath the capsule, metastases were not found in any other tissue, and were notably absent from liver, kidney and spleen.

Fig. 2 shows the overall 4h-accumulated mitotic count of the s.c. solid tumour from the time of growth initiation at 2 days until death occurred at 9 days. As the tumour became established in the host and underwent the initial growth phase between 2 and 4 days, a 2-fold increase in cellular proliferation was observed. Thereafter, the 4h-accumulated MI remained constant until the time of death. This corresponded with the period when maximum growth rate was observed.

Insufficient clusters of tumour cells were present in the lungs until 6 days, and in the lymph nodes until 9 days after injection, to warrant counting. At 6 days the 4h-accumulated mitotic count in lung metastases was significantly higher than that observed in the solid s.c. tumour (Fig. 2). A further increase in lung-tumour cellular proliferation was observed at 8 days, but on the 9th day after injection the 4h-accumulated MI fell dramatically.

The 4h-accumulated MI in lymph-node metastases, at 9 days, was significantly higher than that of either the primary tumour or the lung metastases at this stage (Fig. 2). However, the rate of cellular proliferation was comparable in the lymph-node metastases at 9 days and the lung metastases at 8 days, when the peak accumulated MI was observed.

When metastatic cells were counted in the lungs, the microscope fields were categorised according to the presence of lung tissue interspersed with tumour cells, the peripheral 120$\mu$m of the main tumour mass or the central tumour mass.

A gradation of the 4h-accumulated MI was observed across the metastatic nodules (Fig. 3). The highest rate of cellular proliferation was observed in the central

Fig. 3.—The mitotic index of: A, Walker tumour cells interspersed with host lung tissue; B, tumour cells at the periphery of a solid tumour mass within the lung; and C, tumour cells at the centre of the tumour mass within lung. (Results expressed as mean$\pm$s.e.% mitosis).

tumour mass, with a significantly lower rate of mitosis at the periphery of the main tumour mass, and a further significant decrease in tumour-cell mitosis in the presence of lung tissue. These locational differences in the accumulated mitotic count were observed at all 3 sampling times after tumour-cell injection. Unfortunately, the sparsity of tumour-cell clusters made this kind of classification of tumour-cell counts in the lymph nodes impossible.

### DISCUSSION

The present findings using the 4h-accumulated mitotic count as an indicator of cellular proliferation agree with the earlier report of Bertalanffy and Lau (1962) that, following the latent period, no significant variation occurred in cellular proliferation during growth of solid tumours. The tumour grew at a steady rate after adaptation to a new host.

The higher rate of cellular proliferation in the lung and lymph-node metastases, when compared with primary tumours of either the same size or the same time after cell injection, supports the evidence of Kiseleva (1961) and that of Simpson-Herren, Sanford and Holmquist (1974) who showed that the cell cycle and S phase were shorter in Lewis lung carcinoma metastases than in the primary tumour. These findings are consistent with the greater drug sensitivity of lung metastases reported by Trope (1975).

It may be suggested that this difference in cellular proliferation between metastases and primary tumour arises from selection of cells during or after implantation, resulting in a biochemically different phenotype in the metastases. This contention has some support from the work of Trope (1975) who demonstrated increased drug sensitivity in subcutaneous tumours derived from successive passages of the tumour as metastases.

Alternatively, it could be postulated that local factors in the host tissue specifically influence tumour proliferation. This may take the form of a more adequate blood supply and better nutrition in the metastases than in the primary tumour. The fact that necrotic areas were never observed in metastatic nodules supports this idea.

Our findings of a higher rate of cellular proliferation in the central mass of the metastases than in the peripheral areas may also be explained by regional differences in blood supply to the metastases. It may be that the established core of the tumour nodules is particularly well vascularised, compared with the periphery of the nodule. This rich vascularization may result in a higher rate of cellular proliferation near the capillaries (Tannock, 1970) as well as the removal of toxic metabolites, which may be one of the causes of necrosis and inhibition of cell division (Bröyn, 1975*b*).

However, there is increasing evidence of humoral factors, emanating from the host tissue, which influence tumour growth (Billingham and Silvers, 1971; Bröyn, 1975*a*; Lowenstein and Penn, 1967; Van Scott and Reinertson, 1961). Thus, the possibility that host lung tissue exerts some inhibitory influence on the development of metastases cannot be ignored. If this is the case, the gradation of this influence into the solid tumour mass indicates that this is not a cell-contact phenomenon, but rather supports the theory of diffusible humoral factor. In addition it may be tentatively suggested that differences in the effectiveness of host inhibitors may be involved in different site patterns of tumour growth.

The expert technical assistance of Miss A. Franklin and Mr A. Stevenson is gratefully acknowledged.

### REFERENCES

Baserga, R., Kisieleski, W. E. & Halvorsen, K. (1960) A Study on the Establishment and Growth of Tumour Metastases. *Cancer Res.*, **20**, 910.

Bertalanffy, F. D. & Lau, C. (1962) Rates of Cell Division of Transplantable Malignant Rat Tumours. *Cancer Res.*, **22**, 627.

Billingham, R. E. & Silvers, W. K. (1971) A Biologist's Reflections on Dermatology. *J. invest. Derm.*, **57**, 227.

Bröyn, T. (1975*a*) The Interaction Between Walker Tumour Cells and Mucosa cells in the Lamina Propria of Gastric Mucosa in Rats. *Virchows Arch. B Cell Path.*, **19**, 27.

Bröyn, T. (1975*b*) Kinetics of Cell Proliferation and Cell Loss in the Peripheral and Central Parts of Walker Tumours Growing in Rats and Nude Mice. *Virchows Arch. B Cell Path.*, **18**, 181.

Carr, I., Norris, P. & McGinty, F. (1975) Reverse Diapedesis, the Mechanisms of Invasion of Lymphatic Vessels by Neoplastic Cells. *Experientia*, **31**, 590.

Foulds, L. (1969) *Neoplastic Development*, Vol. 1. London: Academic Press Inc.

Kiseleva, E. G. (1961) Autoradiographic Studies of Walker Carcinosarcoma and its Metastases. *Vops. Onkol.*, **19**, 86.

Lowenstein, W. R. & Penn, R. D. (1967) Intercellular Communication and Tissue Growth. *J. Cell Biol.*, **33**, 235.

Marrable, A. W. (1962) The Counting of Cells and Nuclei on Microtome Sections. *Quart. J. micr. Sci.*, **103**, 331.

Saidel, G. M., Liotta, L. A. & Kleinerman, J. (1976) System Dynamics of a Metastic Process from an Implanted Tumour. *J. theor. Biol.*, **56**, 417.

Simpson-Herren, L., Sanford, A. H. & Holmquist, J. P. (1974) Cell Population Kinetics of Transplantable and Metatastic Lewis Lung Carcinoma. *Cell Tissue Kinetics*, **7**, 349.

Tannock, I. F. (1970) Population Kinetics of Carcinoma Cells, Capillary Epithelial Cells and Fibroblasts in a Transplanted Mouse Mammary Tumor. *Cancer Res.*, **30**, 2470.

Trope, C. (1975) Different Sensitivity to Cytostatic Drugs of Primary Tumor and Metastases of Lewis Carcinoma. *Neoplasma*, **22**, 171.

van Scott, E. J. & Reinertson, R. P. (1961) The Modulating Influence of Stromal Environment on Epithelial Cells Studied in Human Autotransplants. *J. invest. Derm.*, **36**, 109.